# Towards the quantitative characterisation of piglets’ robustness to weaning: a modelling approach

**DOI:** 10.1017/S1751731119000843

**Published:** 2019-05-16

**Authors:** M. Revilla, N. C. Friggens, L. P. Broudiscou, G. Lemonnier, F. Blanc, L. Ravon, M. J. Mercat, Y. Billon, C. Rogel-Gaillard, N. Le Floch, J. Estellé, R. Muñoz-Tamayo

**Affiliations:** 1 GABI, INRA, AgroParisTech, Université Paris-Saclay, 78350, Jouy-en-Josas, France; 2 UMR MoSAR, INRA, AgroParisTech, Université Paris-Saclay, 75005, Paris, France; 3 UE GenESI, INRA, 17700, Surgères, France; 4 IFIP-Institut du porc and Alliance R&D, 35651, Le Rheu, France; 5 UMR PEGASE, INRA, AgroCampus Ouest, 35590, Saint-Gilles, France

**Keywords:** body weight, dynamic modelling, perturbation, resilience, pigs

## Abstract

Weaning is a critical transition phase in swine production in which piglets must cope with different stressors that may affect their health. During this period, the prophylactic use of antibiotics is still frequent to limit piglet morbidity, which raises both economic and public health concerns such as the appearance of antimicrobial-resistant microbes. With the interest of developing tools for assisting health and management decisions around weaning, it is key to provide robustness indexes that inform on the animals’ capacity to endure the challenges associated with weaning. This work aimed at developing a modelling approach for facilitating the quantification of piglet resilience to weaning. A total of 325 Large White pigs weaned at 28 days of age were monitored and further housed and fed conventionally during the post-weaning period without antibiotic administration. Body weight and diarrhoea scores were recorded before and after weaning, and blood was sampled at weaning and 1 week later for collecting haematological data. A dynamic model was constructed based on the Gompertz–Makeham law to describe live weight trajectories during the first 75 days after weaning, following the rationale that the animal response is partitioned in two time windows (a perturbation and a recovery window). Model calibration was performed for each animal. Our results show that the transition time between the two time windows, as well as the weight trajectories are characteristic for each individual. The model captured the weight dynamics of animals at different degrees of perturbation, with an average coefficient of determination of 0.99, and a concordance correlation coefficient of 0.99. The utility of the model is that it provides biologically meaningful parameters that inform on the amplitude and length of perturbation, and the rate of animal recovery. Our rationale is that the dynamics of weight inform on the capability of the animal to cope with the weaning disturbance. Indeed, there were significant correlations between model parameters and individual diarrhoea scores and haematological traits. Overall, the parameters of our model can be useful for constructing weaning robustness indexes by using exclusively the growth curves. We foresee that this modelling approach will provide a step forward in the quantitative characterisation of robustness.

## Implications

The quantitative characterisation of animal robustness at weaning is a key step for management strategies to improve health and welfare. This characterisation is also instrumental for the further design of selection strategies for productivity and robustness. Within a precision livestock farming optic, this study develops a mathematical modelling approach to describe the body weight of piglets from weaning onwards with the rationale that weight trajectories provide central information to quantify the capability of the animal to cope with the weaning disturbance.

## Introduction

In modern swine breeding conditions, weaning is one of the most critical phases (Lallès *et al.*, 2007) because it constitutes a sudden, short and complex event characterised by changes in diet, social and environmental conditions (Campbell, *et al.*, 2013; Blavi, *et al.*, 2016). Moreover, it usually occurs around 3 to 4 weeks in swine commercial conditions after birth although natural weaning lasts up to 17 weeks after birth (Jensen, *et al.*, 1986). The switch from highly digestible liquid milk to a less-digestible and more complex solid feed has consequences on the physiology of the gastrointestinal tract, causing a transitory anorexia, intestinal inflammation and unbalanced gut microbiota (Pié *et al.*, 2004). Often, these changes trigger the development of a dysbiotic state of the gut microbiota that can ultimately result in enteric disease and diarrhoea (Gresse *et al.*, 2017). In general, it has been estimated that in commercial conditions piglets lose about 100 to 250 g BW the first day after weaning regardless of weaning age (Le Dividich and Sève, 2000). For this reason, during this period, the prophylactic use of antibiotics is still widespread to limit piglet morbidity and diarrhoea episodes. However, this procedure raises economic and public health concerns, such as the growing number of antimicrobial-resistant agents. In this context, finding antibiotic alternatives to maintain piglet health at the critical weaning period and preserve public health becomes a high priority. Consequently, there is an increased interest to develop tools for assisting health (European Medicines Agency, 2017) and management decisions around this critical period.

The response of a piglet to weaning relates to its robustness, that is, its capacity to maintain productivity in a wide range of environments without compromising reproduction, health and welfare (Friggens *et al.*, 2017). A key component of robustness, sometimes termed resilience, is the ability to cope with environmental perturbations (abrupt separation from the sow, a different food source, social hierarchy stress, a different physical environment). One way to characterise resilience is by quantifying the extent of deviations from the non-perturbed trajectories of physiological functions (Codrea *et al.*, 2011). In this respect, the development of mathematical models in animal science can be helpful to gaining insight in animal robustness (e.g. Sadoul, *et al.*, 2015). The Gompertz model (Gompertz, 1825) is well known and widely used to describe the growth of animals (Schinckel *et al.*, 2004). However, the Gompertz model does not account for the effects caused by a perturbation. To consider the effect of a disturbing environment, William Makeham extended the Gompertz model by adding a constant term to explain that the rate of change is also driven by factors that are independent of age (Makeham, 1873). The resulting equation is known as the generalised Gompertz–Makeham equation (Golubev, 2009). Based on this scenario, the aim of the present study was to develop a modelling methodology for quantification of piglet resilience at weaning. A perturbed model was developed to describe animal growth based on the Gompertz–Makeham equation. The model parameters have biological interpretation and inform on the amplitude and length of the perturbation. After defining these biological parameters, correlation analyses with faecal score data and haematological traits were performed in order to evaluate the pertinence of these parameters for assessing differences in robustness.

## Material and methods

### Animal samples

The animal resource population used in this study comes from *Institut National de la Recherche Agronomique’s* (**INRA**) experimental farm (*Le Magneraud*, France). Here, results based on 325 piglets from a French Large White selected line (Tribout *et al.*, 2010) within the Pigletbiota ANR project are reported. All animal procedures were performed according to the guidelines for the care and use of experimental animals established by INRA (Ability for animal experimentation to J. Estellé: R-94ENVA-F1-12; agreement for experimentation of INRA’s *Le Magneraud* farm: A17661; protocol approved by the French Ministry of Research with authorisation ID APAFIS#2073-2015092310063992 v2 after the review of ethics committee nº084).

Piglets were weaned at an average age of 28.66 ± 1.17 days (± standard error of the mean; **SEM**) and weighed 8.91 ± 0.49 kg. All animals were maintained under standard intensive conditions and feeding was *ad libitum* with two cereal-based pellet standard diets formulated to exceed the nutrient requirements of the animals. Nursery diet provided 10.5 MJ/kg of net energy for production with 17.5% of crude protein, 1.2% of lysine and included sour whey and extruded cereal and soya. Growing diet provided 9.7 MJ/kg of net energy for production with 17.3% of crude protein and 1.05% of lysine. The management, environmental and housing factors were identical for the three batches of animals during the whole study. None of the animals received antibiotic treatments during the study and the animals were free of the principal swine infectious agents.

### Measures of animal’s body weight

Body weight data were collected at birth, then three times before the weaning period (once per week), two times per week from 29 to 50 days, once per week from 50 to 100 days, and then once every 2 weeks until the end of the animal’s life. On average, each pig was weighed 20 times. The measures of animal’s BW were carried out with two different scales: a SWR3P1 (Balea Group, Saint-Mathieu-de-Tréviers, France) scale (with a BW limit of 30 kg) for the nursery, and a SWR3P-BMC scale (with a BW limit of 150 kg) for the fattening period.

### Faecal score data

Faeces were individually observed at days 0, 2, 6, 8, 12, 15, 20, 27 and 34 with respect to weaning and were scored according to three levels: 0 for normal, 1 for soft faeces but without diarrhoea and 2 for evident diarrhoea (Le Floc’h *et al.*, 2014). The faecal score data were analysed in three different ways: (1) by summing the number of diarrhoea measurements and correcting by the number of observations per animal (FS_sum); (2) by group levels, being level 0 for those animals that have no diarrhoea observations on any of the measures, level 1 for those that have only one diarrhoea record and level 2 for the animals that have shown two or more diarrhoea records (FS_gr); and (3) by the presence or absence of diarrhoea records (FS_p_a) taking into account all the observations made.

### Blood sampling and haematological traits

Blood samples were collected at 28 and 34 days of age from the jugular vein into 4 mL vacutainer tubes containing ethylenediaminetetraacetic acid as anticoagulant.

The haematological analyses were carried out within a few hours after sampling, using a MS9-5 instrument (Melet Schloesing, Osny, France). The haemograms included: basophils (**Bas**) [%], blood plate (**Plt**) [m/mm^3^], eosinophils (**Eos**) [%], erythrocytes (**Ery**) [m/mm^3^], haematocrit (**Hct**) [%], haemoglobin (**Hgb**) [g/dl], leucocytes (**Leu**) [m/mm^3^], lymphocytes (**Lym**) [%], mean corpuscular haemoglobin concentration (**MCHC**) [g/dl], mean corpuscular haemoglobin content (**MCH**) [pg], mean corpuscular volume (**MCV**), monocytes (**Mon**) [%] and neutrophils (**N**) [%]. In addition, the neutrophils/lymphocyte ratio (**N/Lym**) [%] was estimated as a measure of stress (Puppe *et al.*, 1997). These measurements were recorded as important indicators of health and disease in animals. The reference values of these haematological measurements (Radostits *et al.*, 2006) vary according to age, sex, breed, sampling technique and testing methodology. As such, the range limits are not firm boundaries and should be used as guidelines (Humann-Ziehank and Ganter, 2012).

### Mathematical modelling of growth with the Gompertz function

The global dynamics of many natural processes including growth have been described by the Gompertz function (Waliszewski and Konarski, 2005). The essential characteristic of this function is that it exhibits an exponential decay of relative growth rate, making this model a reference for describing the growth of many types of organisms. As a first step for quantifying robustness, we focused on the dynamics of non-perturbed live weight during the first 75 days after weaning using the Gompertz equation (1) using the formulation of Schulin-Zeuthen *et al.* (2008). (1)
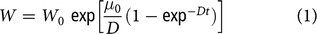
where *W*_0_ is the initial value of live weight *W* (kg), *µ*_0_ is the initial value of the specific growth rate *µ* (d^−1^), the constant *D* (d^−1^) is a growth rate coefficient that controls the slope of the growth rate curve and *t* (days) is time since weaning.

### Perturbed growth modelling with the Gompertz–Makeham function

The Gompertz model is a monotonic function that does not account for possible decrease of weight gain due to perturbations. To elaborate further on our hypothesis to quantify robustness at weaning period, we took as a basis the Gompertz–Makeham extension (Golubev, 2009) that has the advantage of explicitly including the perturbation and thus allows to describe weight gain decrease. The weight dynamics is described by the following model with two ordinary differential equations: (2)
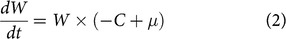

(3)
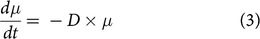
where *C* (d^−1^) is a parameter representing the effect of the environment on the weight change. Note that if *C* = 0, the equations (2) and (3) are the differential form of the classic Gompertz equation. Equations (2) and (3) can be arranged into one equation.(4)
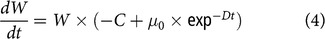



In the remaining of the text, we refer to the model in Eq. (4) as the perturbed growth model. The specificity of our approach relies on the hypothesis that the weight dynamics of the animal is partitioned into two time windows (perturbed and recovery windows) to represent the moment at which the animal is perturbed and the moment at which it recovers from the perturbation. This is translated mathematically by modulating the parameter *C* with the following conditions: (5)
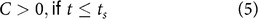

(6)
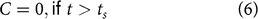
where *t_s_* (days) is the time of the recovery switch that is assumed to be specific for each animal.

### Model evaluation and calibration

We tested the structural identifiability of both the Gompertz and the perturbed models. That is, we determined if it was theoretically possible to determine uniquely the model parameters given the available measurements (see, e.g. Muñoz-Tamayo *et al.* (2018) for a discussion on structural identifiability). Identifiability testing was performed using the freely available software DAISY (Bellu *et al.*, 2007). Both models are structurally globally identifiable, implying that the parameter estimation problem is well posed (it has unique solution).

The mathematical models (Gompertz and perturbed) were further calibrated for each animal by minimising the least squares error: (7)
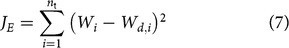
where *W_d_* is the weight data (kg), *W* the weight predicted by the model and *n*_t_ the total number of measurements. For the Gompertz model, the parameters to be estimated from the calibration were *D* and *µ*_0_. For the Gompertz–Makeham equation, the parameters to be estimated from the calibration were *D*, *µ*_0,_ *C* and *t_s_*. The numerical estimation of the parameters was performed in Scilab using the Nelder–Mead algorithm implemented in the ‘*fminsearch*’ function (Scilab Enterprises, 2012) (v.6.0.0).

To allow comparison between all animals within the studied population, for each individual the objective function was further weighted with respect to the number of measurements for each animal (*n*_t_).(8)
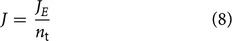



In addition, we carried out a calibration of the Gompertz model using the BW at weaning and only the last four records during the first 75 days after weaning of each animal (*n_t_* = 5). We assumed that the resulting model calibrated with these data is an approximation of the theoretical growth rate of the animals not experiencing any perturbation. In the remaining text, we will call the trajectory resulted from this calibration with the subset data as the unperturbed curve.

The Akaike Information Criterion (**AIC**) (Akaike, 1974) was used to select the best candidate model. The AIC is a trade-off between goodness of fitness and model complexity. The AIC was calculated as follows (Banks and Joyner, 2017): (9)
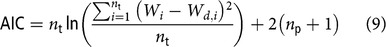
where *n*_p_ is the total number of model parameters. When there are several competing models, the best model in the AIC sense is the one with the smallest AIC.

Model performance was assessed by classical statistical indicators, namely coefficient of determination (*r* ^2^) and the Lin’s concordance correlation coefficient (**CCC**) (Lin, 1989).

### Statistical analyses to relate model parameters with haematological traits

Correlation analyses were performed to explore the relationships between the perturbed growth model parameters and the faecal score data and haematological indicator data. Pearson and Spearman correlations among them were analysed in R using the ‘*cor*’ function in the base package (R Core Team, 2017). When required, data were normalised applying the log_2_ or log_10_ transformation. Visualisation of correlations was performed with the ‘*corrplot*’ R package (Wei and Simko, 2017). Only the correlations with *P*-value less than 0.05 were considered as significant and were thus represented.

## Results

### Growth curve modelling from weaning

**Figure 1:**
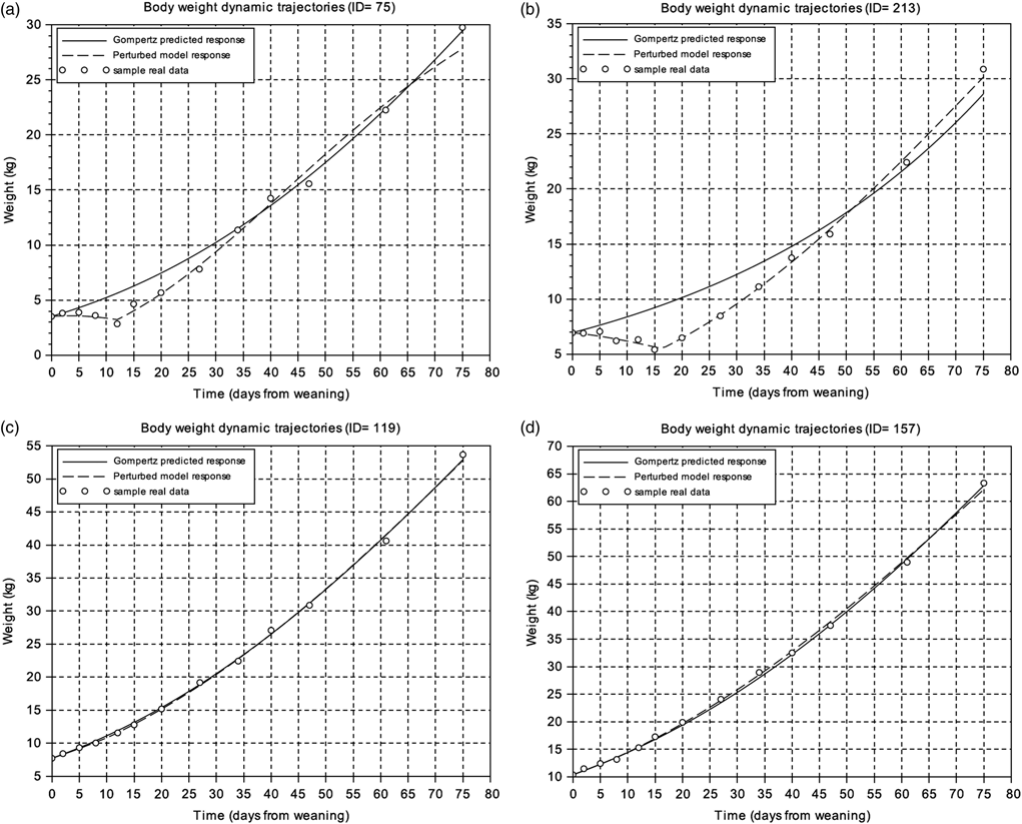
Body weight dynamics trajectories. (a) and (b) represent samples with the worst level of fitting using the Gompertz model. (c) and (d) represent samples with the best fitting using the Gompertz model. Circles represent the different BW measures of the individual piglet relative to days from weaning, the solid line is the Gompertz predicted response and the dashed line is the perturbed growth model response.

To get an overview of the piglets’ response to weaning, the BW measurements of the first 75 days after this critical period were analysed. Figure 1 displays the BW dynamic trajectories of four animals exhibiting two extreme patterns. The experimental data were compared to predicted responses given by both the Gompertz and perturbed growth model. Animals were ranked according to the goodness of fit for the Gompertz equation given by the criterion J in Eq. (8). This ranking provided a first indication on the magnitude of the perturbation and animal resilience, i.e. the higher the value of J, the greater the degree of growth perturbation (Supplementary Table S1). Moreover, the analysis of *J* reflects a difference of one order of magnitude between the most growth-perturbed animal and the least growth-perturbed one. As observed in Figure 1, the perturbed growth model accurately described the weight dynamics of animals with different degrees of perturbation (Table 1; Supplementary Table S1). The minimum and the mean values of the *r* ^2^ coefficient were 0.62 and 0.99, respectively. As well, for the CCC coefficient the minimum value was 0.73 and the mean 0.99 (Supplementary Table S1).

**Table 1:** Descriptive statistics for the parameters of the perturbed growth model in pigs

Perturbed growth model parameters	Range	Mean	SD
*µ*_0_	1.22 × 10^−02^ to 1.11 × 10^−01^	4.78 × 10^−02^	1.22 × 10^−02^
*D*	1.24 × 10^−03^ to 4.25 × 10^−02^	1.53 × 10^−02^	5.57 × 10^−03^
*C*	4.97 × 10^−10^ to 1.00 × 10^−01^	2.84 × 10^−02^	1.47 × 10^−02^
*t_s_*	0.00 to 23.75	9.94	3.41
*J*	3.70 × 10^−05^ to 5.42 × 10^−03^	1.00 × 10^−03^	7.79 × 10^−04^
*ABC*	0.00 to 137.86	29.72	22.96

*µ*_0_ = individual growth rate at the moment of weaning (d^−1^);*D* = extent of the exponential decay of the growth (d^−1^); *C* = constant related to the level of perturbation (d^−1^); *t_s_* = moment at which the animal recover for the perturbation (days); *J* = model error (kg^2^); *ABC* = area between curves; SD = standard deviation.

According to the AIC criterion, the perturbed growth model was better than the Gompertz model. In addition, the advantage of the perturbed growth model is that it provides biologically relevant parameters that inform on the amplitude (degree of the perturbation) and length of perturbation, and the rate of animal recovery. The parameter *µ*_0_ represents the individual growth rate at the moment of weaning. The parameter *C* determines the degree of perturbation: the higher the value of this parameter, the higher the growth perturbation of the animal. The parameter *t_s_* indicates the moment at which the animal starts to recover from the perturbation (from our results, it could be extracted that 9 days is the time that a pig lasts to recover in average from the perturbation of weaning). Finally, the parameter *D* indicates the exponential rate of decay of growth rate. The descriptive statistics and the estimated parameter values of the perturbed growth model are given in Table 1 and Supplementary Table S1, respectively.

**Figure 2:**
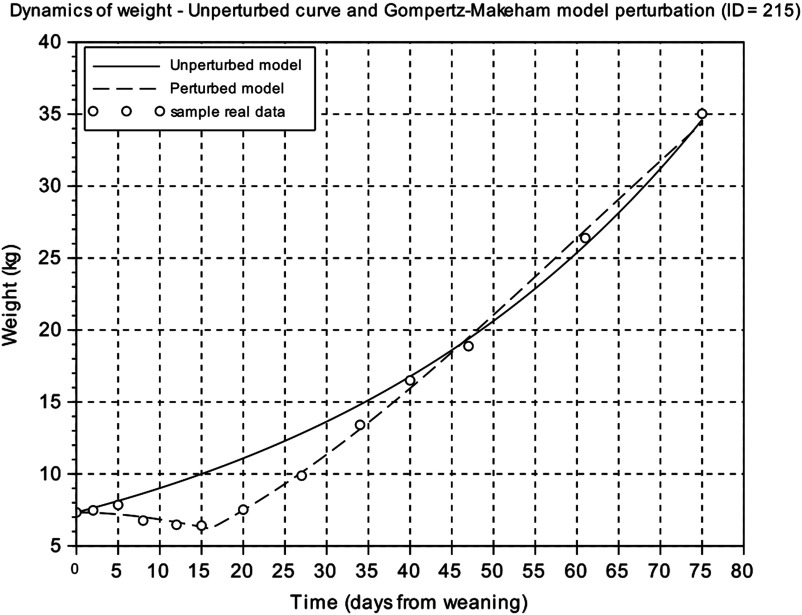
Comparison of the weight dynamics as predicted by the unperturbed and the perturbed (Gompertz–Makeham) models. Animal ID=215 is represented. Circles represent the different BW measures of the individual piglet relative to days from weaning, the solid line is the predicted response of the unperturbed growth model and the dashed line is the perturbed growth model response.

From the unperturbed growth curve for each animal obtained from the calibration of the Gompertz model using the BW at weaning and the last four records during the first 75 days after weaning of each animal, we calculated the area between the unperturbed growth curve and the perturbed growth curve from weaning to the time where the curves intersect. The resulting value was called the area between curves (***ABC***) index and reflects the difference between the unperturbed growth curve and the perturbed growth model response (Figure 2) (Supplementary Table S1).

**Figure 3:**
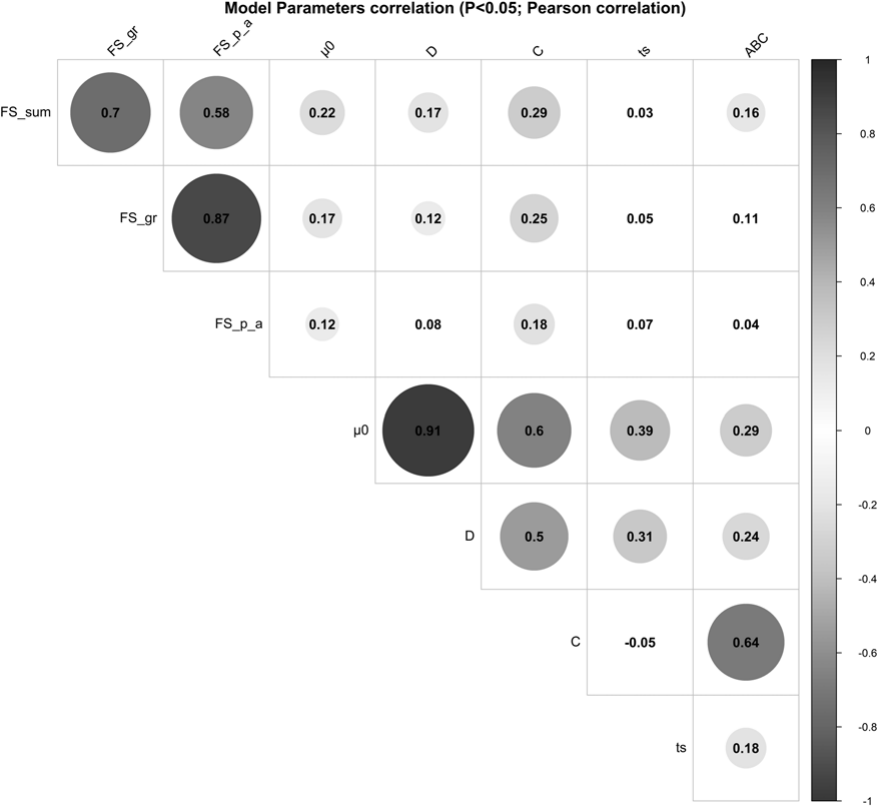
Pearson’s coefficients to visualise correlations among the model parameters of the Gompertz–Makeham perturbed growth model in pigs and the faecal score data. The size of the circles is proportional to the correlation coefficients. Only the correlations with *P*-value less than 0.05 were considered as significant and were represented with circles, and the insignificant correlations are left blank. Faecal score data, analysed as a continuous variable (FS_Sum), by groups (FS_gr), and by the presence/absence (FS_p_a). *µ*_0_ (d^−1^): individual growth rate at the moment of weaning; *D* (d^−1^): rate coefficient controlling the slope of the growth rate *µ*; C (d^−1^): coefficient representing the effect of the perturbation; *t_s_* (d): time at which the animal recover for the perturbation; *ABC:* area between the unperturbed and perturbed model curves.

**Figure 4:**
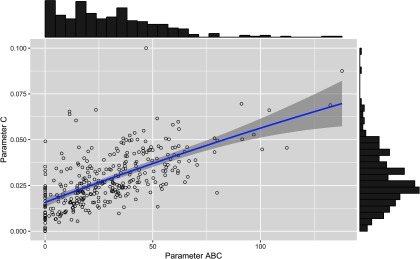
Scatter plot with marginal histograms illustrating the relationship between parameter *C* (level of perturbation) and parameter *ABC* (area between the unperturbed and perturbed model curves) of the perturbed growth model in pigs.

The parameter *µ*_0_ showed significant positive correlations with parameter *D* (*r* = 0.91, *P* < 0.001), parameter *C* (*r* = 0.60, *P* < 0.001), parameter *t_s_* (*r* = 0.39, *P* < 0.001) and parameter *ABC* (*r* = 0.29, *P* < 0.001). A moderate-to-strong significant positive correlation was estimated between the parameters *C* and *ABC* of the perturbed growth model (*r* = 0.64, *P* < 0.001) (Figure 3). There was an obvious correlation for those animals that had a greater degree of growth perturbation to be associated with a higher *ABC* parameter (Figure 4). In fact, the parameter *C* is a measure of the deviation between the unperturbed and the perturbed curves. A moderate positive correlation value was found between the parameter *C* and parameter *D* (*r* = 0.50, *P* < 0.001). In contrast, no significant correlation between parameter *C* and parameter *t_s_* was identified. Moreover, parameter *D* showed a positive correlation with the parameter *t_s_* (*r* = 0.31, *P* < 0.001). The weakest significant positive correlation was observed between parameter *D* and parameter *ABC* (*r* = 0.24, *P* < 0.001), and parameter *t_s_* and parameter *ABC* (*r* = 0.18, *P* < 0.001).

### Post-weaning diarrhoea scores and model parameters

The relationships between the model parameters and the faecal score data of weaned piglets were analysed by correlation analyses (Figure 3). Several significant positive correlations were identified. Faecal score data, analysed as a continuous variable (FS_Sum), was positively correlated to the parameter *C* (*r* = 0.29, *P* < 0.001), parameter *µ*_0_ (0.22, *P* < 0.001), parameter *D* (*r* = 0.17, *P* < 0.01) and parameter *ABC* (*r* = 0.16, *P* < 0.01). Moreover, the faecal score data analysed by groups (FS_gr; level 0 = no diarrhoea observations; level 1 = one diarrhoea record; level 2 = two or more diarrhoea records) and the parameter *C*, parameter *µ*_0_ and parameter *D* had a correlation coefficient of 0.25 (*P* < 0.001), 0.17 (*P* < 0.01) and 0.12 (*P* < 0.05), respectively. The presence/absence (FS_p_a) of diarrhoea records showed significant correlations with parameter *C* (*r* = 0.18, *P* < 0.01) and parameter *µ*_0_ (*r* = 0.12, *P* < 0.05).

### Blood cell population and model parameters

To evaluate piglet health status at weaning period, several blood cell measurements were recorded at weaning (28 days) and 1 week later (34 days).

The correlation analyses performed between the available haematological measurements of the 320 animals at 34 days, and the model parameters showed positive and negative significant correlations (Table 2).

The most significant correlations were between the parameter *D* and Hgb (*r* = −0.23, *P* < 0.001), MCH (*r* = −0.22, *P* < 0.001) and Plt (*r* = 0.22, *P* < 0.001).

**Table 2:** Pearson’s coefficients to visualise correlations among the model parameters of the Gompertz–Makeham perturbed growth model and the haematological measurements (34 days) (n = 320 pigs)

	*D*	*C*	*t_s_*	*ABC*	BW_w	Age_w	Leu	Lym	Mon	N	Eos	Bas	Ery	MCV	Hct	MCH	MCHC	Hgb	Plt	N/Lym
*µ*_0_	0.90***	0.52***	0.43***	0.30***	−0.29***	0.19***	0.03	−0.12*	−0.02	0.13*	0.11	−0.04	−0.03	−0.05	−0.06	−0.11	−0.15**	−0.12*	0.16**	0.11
*D*		0.43***	0.40***	0.24***	−0.05	0.25***	0.07	−0.09	−0.08	0.10	0.15**	0.01	−0.03	−0.18***	−0.18**	−0.22***	−0.15**	−0.23***	0.22***	0.09
*C*			0.00	0.57***	0.03	0.13*	−0.12*	0.01	−0.12*	0.02	0.13*	−0.10	0.14*	−0.16**	−0.03	−0.18**	−0.09	−0.07	0.13*	−0.01
*t_s_*				0.25***	−0.15**	0.13*	−0.16**	0.00	−0.11	0.02	0.08	−0.08	0.04	−0.05	−0.02	−0.07	−0.05	−0.04	0.06	0.03
*ABC*					0.22***	0.24***	−0.11*	−0.02	−0.30***	0.08	0.20***	−0.16**	0.15**	−0.18***	−0.04	−0.16**	−0.01	−0.05	0.03	0.04

*µ*_0_ = individual growth rate at the moment of weaning (d^−1^);*D* = extent of the exponential decay of the growth (d^−1^); *C* = level of perturbation (d^−1^); *t_s_* = moment at which the animal recover for the perturbation (days); *ABC* = area between curves; BW_w = BW at weaning (kg); Age_w = age at weaning (days); Leu = leucocytes (m/mm^3^); Lym = lymphocytes (%); Mon = monocytes (%); N = neutrophils (%); Eos = eosinophils (%); Bas = basophils (%); Ery = erythrocytes (m/mm^3^); MCV = mean corpuscular volume; Hct = haematocrit (%); MCH = mean corpuscular haemoglobin content (pg); MCHC = mean corpuscular haemoglobin concentration (g/dl); Hgb = haemoglobin (g/dl); Plt = blood plate (m/mm^3^); N/Lym = neutrophils/lymphocyte ratio (%).

Superscripts refer to probability levels for significance tests (*P < 0.05, **P < 0.01, ***P < 0.001).

There were also moderately significant correlations between the parameter *ABC* and the rate of Mon (*r* = −0.30, *P* < 0.001) and Eos (*r* = 0.20, *P* < 0.001).

Furthermore, the 213 animals with available haematological measurements at 28 days were analysed to explore their association with the model parameters (Table 3). Parameter *ABC* showed the highest number of significant correlations, the strongest being with Hct (*r* = −0.38, *P* < 0.001), Hgb (*r* = −0.32, *P* < 0.001), MCHC (*r* = 0.32, *P* < 0.001), MCV (*r* = −0.32, *P* < 0.001) and Ery (*r* = −0.20, *P* < 0.01).

**Table 3:** Pearson’s coefficients to visualise correlations among the model parameters of the Gompertz–Makeham perturbed growth model and the haematological measurements (28 days) (n = 213 pigs)

	*D*	*C*	*t_s_*	*ABC*	BW_w	Age_w	Leu	Lym	Mon	N	Eos	Bas	Ery	MCV	Hct	MCH	MCHC	Hgb	Plt	N/Lym
*µ*_0_	0.88***	0.49***	0.41***	0.42***	−0.18**	0.30***	0.02	0.12	−0.13	−0.09	0.15*	−0.11	0.01	−0.13	−0.08	−0.03	0.15*	−0.02	−0.04	−0.10
*D*		0.40***	0.33***	0.37***	0.07	0.36***	0.05	0.18**	−0.20**	−0.14*	0.18**	−0.12	−0.05	−0.22**	−0.19**	−0.12	0.16*	−0.16*	0.04	−0.15*
*C*			0.03	0.63***	0.11	0.24***	−0.13	0.06	−0.12	−0.02	0.02	−0.15*	−0.11	−0.23***	−0.24***	−0.12	0.18**	−0.21**	0.03	−0.04
*t_s_*				0.36***	−0.16*	0.09	−0.10	0.02	−0.01	−0.01	0.01	−0.05	0.10	−0.02	0.07	0.02	0.06	0.11	0.02	−0.03
*ABC*					0.27***	0.39***	−0.12	0.14*	−0.14*	−0.11	0.09	−0.19**	−0.20**	−0.32***	−0.38***	−0.13	0.32***	−0.32***	0.17*	−0.14*

*µ*_0_ = individual growth rate at the moment of weaning (d^−1^);*D* = extent of the exponential decay of the growth (d^−1^); *C* = level of perturbation (d^−1^); *t_s_* = moment at which the animal recover for the perturbation (days); *ABC* = area between curves; BW_w = BW at weaning (kg); Age_w = age at weaning (days); Leu = leucocytes (m/mm^3^); Lym = lymphocytes (%); Mon = monocytes (%); N = neutrophils (%); Eos = eosinophils (%); Bas = basophils (%); Ery = erythrocytes (m/mm^3^); MCV = mean corpuscular volume; Hct = haematocrit (%); MCH = mean corpuscular haemoglobin content (pg); MCHC = mean corpuscular haemoglobin concentration (g/dl); Hgb = haemoglobin (g/dl); Plt = blood plate (m/mm^3^); N/Lym = neutrophils/lymphocyte ratio (%).

Superscripts refer to probability levels for significance tests (*P < 0.05, **P < 0.01, ***P < 0.001).

Regarding the parameter *D*, it should be emphasised that it was significantly negatively associated with MCV (*r* = −0.22, *P* < 0.01) and Mon (*r* = −0.20, *P* < 0.01).

In the case of parameter *C*, the strongest negative significant associations were with Hct (*r* = −0.24, *P* < 0.001), MCV (*r* = −0.23, *P* < 0.001) and Hgb (*r* = −0.21, *P* < 0.01).

## Discussion

The challenge to obtain reliable estimates of weaning robustness in large populations motivated us to investigate the usefulness of a modelling approach based on piglet growth curves. Here we describe how we achieved this objective by modelling perturbed growth using the Gompertz–Makeham function. This model successfully characterises growth resilience based on the dynamics of live weight, which could be recorded in swine production systems. This would facilitate its future deployment in pig breeding systems. In addition, the implementation of the approach is made available via the SciLab open-source numerical computation package.

### The Gompertz–Makeham function efficiently models the growth perturbation at weaning

Previous studies evaluating different functions for describing growth in pigs (Schulin-Zeuthen *et al.*, 2008) showed the bounties of the most used functions to represent changes in BW. Due to the practical implications of characterising growth in pigs, different models have been developed to represent growth dynamics (Schulin-Zeuthen *et al.*, 2008).

To our knowledge, this is the first report in modelling the live weight response of piglets’ at weaning. Various statistical and reliability measures of the model are obtained related to the post-weaning growth perturbation, including parameters related to the depth, to the decay of the growth and to the recovery time derived from this perturbation. The application to the population data demonstrates that these parameters appear close to normal distribution. Our study suggests that this model development is instrumental to define parameters for evaluating the level of robustness of animals in current swine production systems.

### Applicability of the model in porcine production

Since more farms invest in precision livestock technologies, such as a weight collecting systems (Stygar *et al.*, 2018), the opportunity of deploying modelling approaches into real-life situations becomes more feasible. Our study developed a mathematical model to describe piglet robustness at weaning by using the live weight during the first 75 days after weaning. We introduce here five weaning robustness parameters that have shown correlations to relevant haematological traits at weaning: *µ*_0_,*C*, *D*, *t_s_* and *ABC*. The first one, parameter *µ*_0_, characterises the individual growth rate at the moment of weaning. Parameter *C* is a measure of the degree of growth perturbation. The practical use of parameter *C* as a measure of the perturbation relies on the idea that the greater the perturbation, the higher growth sensitivity of the animal to weaning stress. This parameter should be interpreted together with the parameter *D*, which indicates the extent of the exponential decay of growth due to the perturbation. In this context, *D* reflects growth robustness rather than resilience to the weaning perturbation. In the perturbed growth model, the animal response to the perturbation is partitioned into a perturbation and a recovery window, with transition time being defined by the parameter *t_s_*. As with *C*, the *t_s_* parameter would be related to the concept of resilience, as a lower *t_s_* would represent an animal that is recovering sooner from the perturbation.

In an attempt to create a measure that summarises the fitted model parameters, we have proposed the *ABC* index, which reflects the difference between a theoretical unperturbed growth curve and the perturbed growth model response. We consider that this parameter is a good candidate to represent the global robustness of the animals at weaning, because it informs on the animal capabilities in terms of the amplitude and length of perturbation, and the rate of animal recovery. For a real implementation at large scale in pig breeding systems, using this *ABC* index has great advantages in terms that it provides an indicator of robustness/resilience to weaning. An alternative to explore could be the idea of developing a robustness index integrating the different parameters of the perturbed growth model.

It should be noted that modelling approach presented here is built on the basis of frequent BW measurements, with particular attention to the first weeks after weaning. The limitation of our approach is that to guarantee a robust model fit, a high frequency of animal measurements is required. Nevertheless, this limiting factor should become less important with the growing prevalence of modern farm systems with automatic measurement recording (Kashiha *et al.*, 2014).

### The proposed robustness parameters correlate with diarrhoea score and blood formula

Positive correlations between parameter *C* and the faecal score data were found in the present study. Given that the faecal score is a measure of the prevalence of digestive disorders, this indicates that the perturbed growth model provides useful information on the health component of robustness to weaning. Weaning is usually associated with a dramatic reduction in food intake, resulting in altered structure of the small intestine. Piglets usually showed weight loss during the first 3 days post-weaning. During the first week post-weaning, the reported prevalence of diarrhoea on at least one day is 73% (Vente-Spreeuwenberg *et al.*, 2003).

Blood constitutes a relevant tissue for phenotyping immune capacity (Flori *et al.*, 2011; Mach, *et al.*, 2013; Schroyen and Tuggle, 2015) and evaluating the health status of pigs. To study the health status in piglets during the period after weaning, we conducted correlation analyses between the haematological traits collected at weaning (28 days) and 1 week after weaning (34 days), and the fitted model parameters. The first week after weaning is considered the most stressful period for piglets, when intestinal dysfunction and changes in metabolism occur (Campbell, *et al.*, 2013), as well as changes in physiological and immunological parameters (Pié *et al.*, 2004; Kick, *et al.*, 2012).

With respect to the blood measurements made at weaning (28 days), a moderate positive correlation between the parameter *ABC* and the level of MCHC (*r* = 0.32, *P* < 0.001), and a negative correlation with MCV levels (*r* = −0.32, *P* < 0.001) was found. Some studies have suggested that MCHC and MCV parameters are early indicators of iron deficiency (Svoboba *et al.*, 2008). Additionally, negative correlations were found between the *ABC* parameter and the percentage of Hct (*r* = −0.38, *P* < 0.001) and the levels of Hgb (*r* = −0.32, *P* < 0.001) at 28 days of age. It has been demonstrated that there is a positive association between Hgb, Hct and average daily gain (**ADG**) in the 3 weeks post-weaning period (Bhattarai and Nielsen, 2015). These authors also reported that an increase in 10 g haemoglobin/litre blood corresponded to an improvement of 17.2 g/day ADG. The negative correlations observed in this study between the parameter *ABC* and Hgb and Hct are in agreement with the reported publication, showing that those perturbed animals grow less.

Regarding the blood measurements collected 1 week after the weaning (34 days), the negative correlation between parameter *ABC* and the percentage of Mon (*r* = −0.30, *P* < 0.001) is noteworthy. The percentage of Mon could be a good estimator of animal health status due to its important roles in both innate and adaptive immune responses, killing microbial pathogens and tumour cells, and exerting immunoregulatory functions through cytokine production and processing and presentation of antigens to Lym (Chamorro, *et al.*, 2005). Moreover, it is assumed that pigs are physiologically and immunologically competent by 35 days of age (Kick, *et al.*, 2012).

Whilst promising, the interpretation of the correlations between the proposed resilience and robustness indicators (derived from the growth curve models) and the health status measurements should be interpreted with caution until they can be tested across a broader range of genotypes and managements scenarios.

## Conclusions

This study presents a method to quantify parameters related to piglet weaning robustness. These parameters are derived by modelling piglet BW trajectories from weaning onwards. This work provides biologically relevant parameters that inform on the amplitude and length of perturbation, and the rate of animal recovery. In addition, we have identified significant correlations between the model parameters and individual diarrhoea scores and haematological measurements, which illustrate the usefulness of these parameters as potential components of an integrated robustness index.

## Notes

M. Revilla, 0000-0002-0164-9317

## Software and data repository resources

The Scilab code for calibration of the perturbed growth model is available under request for academic purposes.

## Supplementary material

For supplementary material accompanying this paper visit https://doi.org/10.1017/S1751731119000843. click here to view supplementary material
